# Chylothorax after hepatectomy: a case report

**DOI:** 10.1186/s13256-018-1882-x

**Published:** 2018-11-26

**Authors:** Ryusei Yamamoto, Yasuji Mokuno, Hideo Matsubara, Hirokazu Kaneko, Yozo Sato, Shinsuke Iyomasa

**Affiliations:** 1 Department of Surgery, Yachiyo Hospital, 2-2-7, Sumiyoshi-cho, Anjo-shi, Aichi 446-8510 Japan; 2 Department of Diagnostic and Interventional Radiology, Aichi Cancer Center Hospital, 1-1 Kanokoden, Chikusa-ku, Nagoya, 464-8681 Japan

**Keywords:** Chylothorax, Hepatectomy, Abdominal surgery, Cholangiocarcinoma, Lymphangiography

## Abstract

**Background:**

Chylothorax is the accumulation of chyle within the pleural space. Chylothorax can occur as a complication after multiple different types of surgery, most frequently after thoracic surgery, albeit with an incidence rate of less than 1%. Chylothorax after abdominal surgery is extremely rare, and there are only a few case reports.

**Case presentation:**

A 74-year-old Japanese woman presented with jaundice. She was diagnosed as having hilar cholangiocarcinoma and underwent right hepatectomy, caudate lobectomy, extrahepatic bile duct resection, and lymph node dissection after preoperative percutaneous transhepatic portal vein embolization. Postoperative liver function was normal. She developed chylous ascites on postoperative day 5, for which conservative treatment was initially effective. Dyspnea developed suddenly on postoperative day 42, and she had a massive right pleural effusion and a small amount of ascites. Management with pleural drainage, total parenteral nutrition, and octreotide injections decreased the chylothorax. However, the chylous effusion reaccumulated on postoperative day 57. As conservative treatments ultimately failed, lymphangiography was performed on postoperative day 62. Lymphangiography with Lipiodol (ethiodized oil) revealed extravasation into the pleural space, but the location of the leak was not identified. There was neither obstruction nor dilation of the thoracic duct. A lymphatic leak in her abdominal cavity was not demonstrated. A chest tube was placed after lymphangiography, and the chylothorax was diminished by postoperative day 71. She was discharged on postoperative day 72. Two and a half years after surgery, she is doing well with no evidence of recurrence of either chylothorax or cancer.

**Conclusions:**

Chylothorax can occur after hepatectomy and pleural effusion should raise suspicion for chylothorax. Lymphangiography may be effective for both diagnosis and treatment in the case of chylothorax after hepatectomy.

## Background

Chylothorax is defined as the accumulation of chyle within the pleural space caused by disruption or obstruction of the thoracic duct or its tributaries, and a pleural fluid triglyceride concentration greater than 110 mg/dL supports the diagnosis. A chylous effusion has a high content of triglycerides, lymphocytes, and immunoglobulins. For this reason, prolonged chylothorax causes dyspnea, immunodeficiency, and malnutrition [1]. Chylothorax can be categorized as nontraumatic or traumatic, the latter of which is almost entirely due to surgery, with a combined incidence of trauma or surgery reported at 50% [2]. Disruption of the thoracic duct at any location can cause chylothorax [3]. Surgical procedures around the thoracic duct can disrupt the thoracic duct or its tributaries [4, 5]. Thoracic surgery, including esophagectomy, lung surgery, and cardiac surgery, is a major cause of traumatic chylothorax but its incidence rate is still less than 1%; retroperitoneal surgery in the vicinity of the cisterna chyli, such as aortic surgery, can lead to either chyloperitoneum or chylothorax [6–12]. However, chylothorax after abdominal surgery is extremely rare [2, 13–15]. Here we present a case of chylothorax after hepatectomy for hilar cholangiocarcinoma. We also review the relevant literature.

## Case presentation

**Fig. 1:**
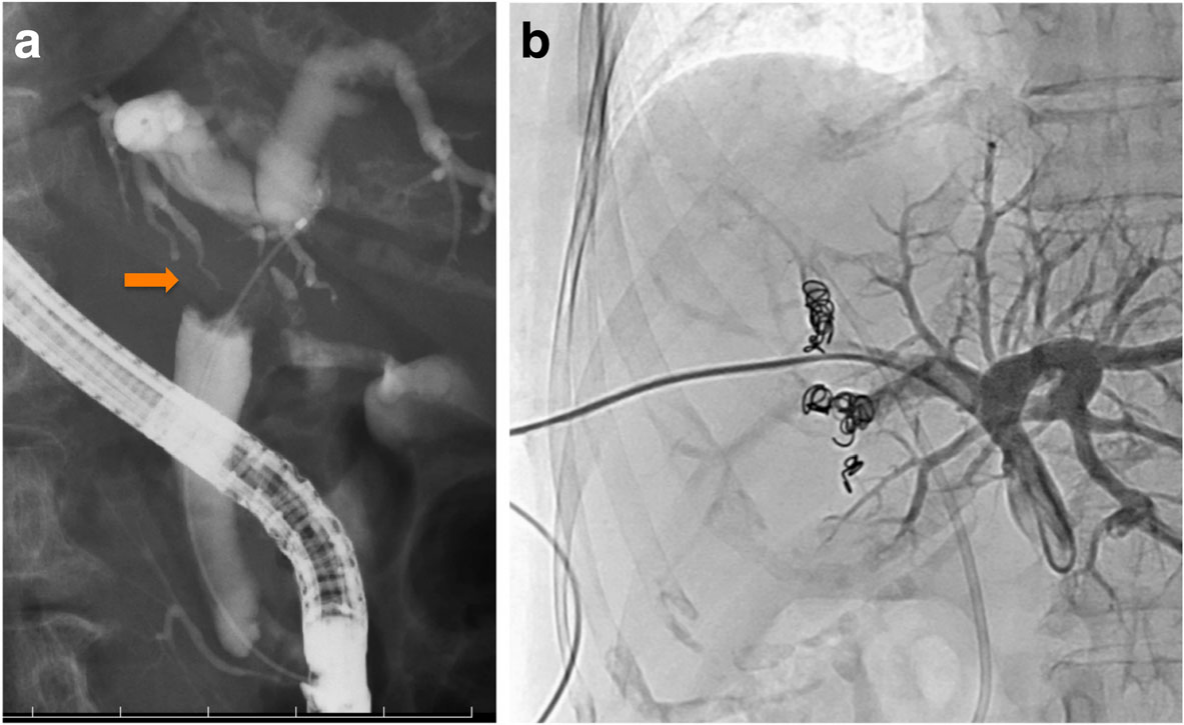
**a** Endoscopic retrograde cholangiopancreatography showing an intraluminal defect in the biliary hilum, Bismuth type 1. The arrow pointing to the intraluminal defect in the biliary hilum. **b** Percutaneous transhepatic portal vein embolization showing the embolization of the right branch of the portal vein performed by puncturing the segment 5a portal vein. Embolization was not done percutaneously through the chest

A 74-year-old Japanese woman was referred to our hospital with a history of several days of jaundice. She had no past medical history and no family history. On examination, her skin was jaundiced, and her abdomen was flat and soft. Her laboratory data revealed obstructive jaundice and cholangitis, and tumor marker levels were elevated with carcinoembryonic antigen at 3.6 and carbohydrate antigen 19-9 at 4573.9. Computed tomography (CT) revealed an enhancing mass in the biliary duct hilum and dilation of the intrahepatic bile ducts, and there was no evidence of lymph node metastasis or distant metastasis. Endoscopic retrograde cholangiopancreatography revealed a luminal filling defect in the biliary hilum (Fig. 1a). We diagnosed the tumor as a Bismuth type 1 cholangiocarcinoma, and performed endoscopic retrograde biliary drainage. Biopsies of the tumor revealed adenocarcinoma. The estimated volume of the postoperative liver remnant was less than 35%. Therefore, percutaneous transhepatic portal vein embolization (PTPE) of the right branch of her portal vein was performed (Fig. 1b). Twenty-one days later, the volume of the left lobe of her liver increased, and we performed right hepatectomy, caudate lobectomy, extrahepatic bile duct resection, and lymph node dissection. Node dissection included resection of hilar and pericholedochal nodes in the hepatoduodenal ligament, common hepatic artery nodes, and those at the celiac trunk, and posterior and anterior pancreaticoduodenal nodes.

**Fig. 2:**
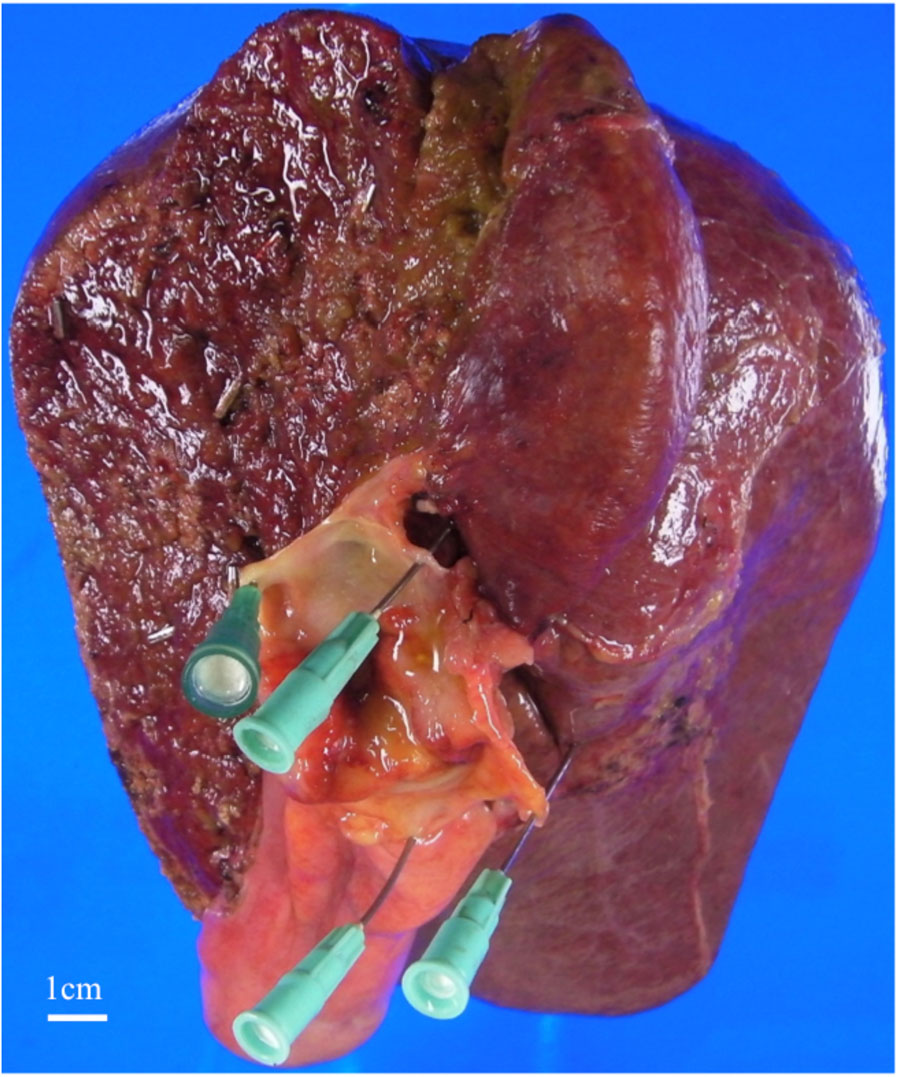
Gross specimen showing hilar cholangiocarcinoma

**Fig. 3:**
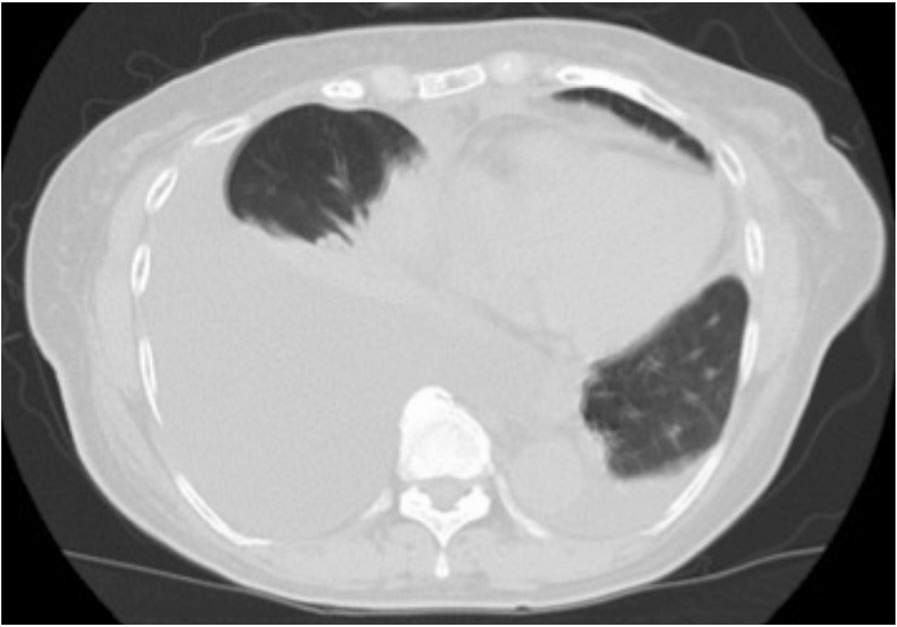
Computed tomography showing large pleural effusion on postoperative day 42

**Fig. 4:**
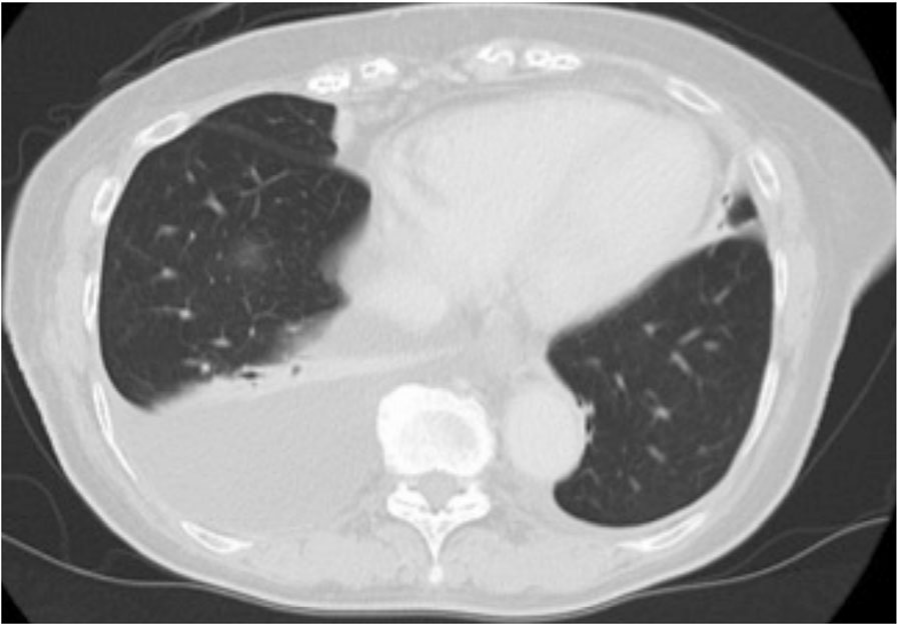
Computed tomography showing reaccumulation of pleural fluid on postoperative day 57

Histological examination of the tumor showed moderately differentiated tubular adenocarcinoma without regional lymph node metastasis, a pathological stage II tumor according to the Union for International Cancer Control classification of malignant tumors, 7th edition (Fig. 2). Postoperative blood laboratory tests showed that liver enzymes were slightly elevated, but that total bilirubin was within normal limits. Resumption of diet started on postoperative day (POD) 3. Although the fluid in the abdominal drain had been serous until POD 4, the appearance of the fluid became milky on POD 5, and the amount of the drainage increased up to 1 L/day. CT showed a large amount of ascites with a small right pleural effusion. We placed our patient on total parenteral nutrition (TPN), and the ascites gradually decreased and became serous again. The abdominal drain was removed on POD 27, and an oral diet was restarted. She experienced sudden dyspnea on POD 42, and CT showed a massive right pleural effusion and a small amount of ascites (Fig. 3). We performed a thoracentesis and placed a chest tube. One liter of chylous effusion was drained, and the triglyceride concentration of the pleural fluid was 1026 mg/dL. With a diagnosis of chylothorax, she was started again on TPN and given subcutaneous octreotide injections. Although the chest drained approximately 2 L/day for several days, both the output and the pleural effusion decreased on chest X-ray. On POD 46, the pleural effusion nearly completely resolved and the chest tube was removed. From then on the octreotide was stopped. Her oral diet was resumed on POD 57, and subsequent CT revealed recurrence of the massive right pleural effusion and a small amount of ascites, leading us once again to make our patient take nil by mouth (Fig. 4).

**Fig. 5:**
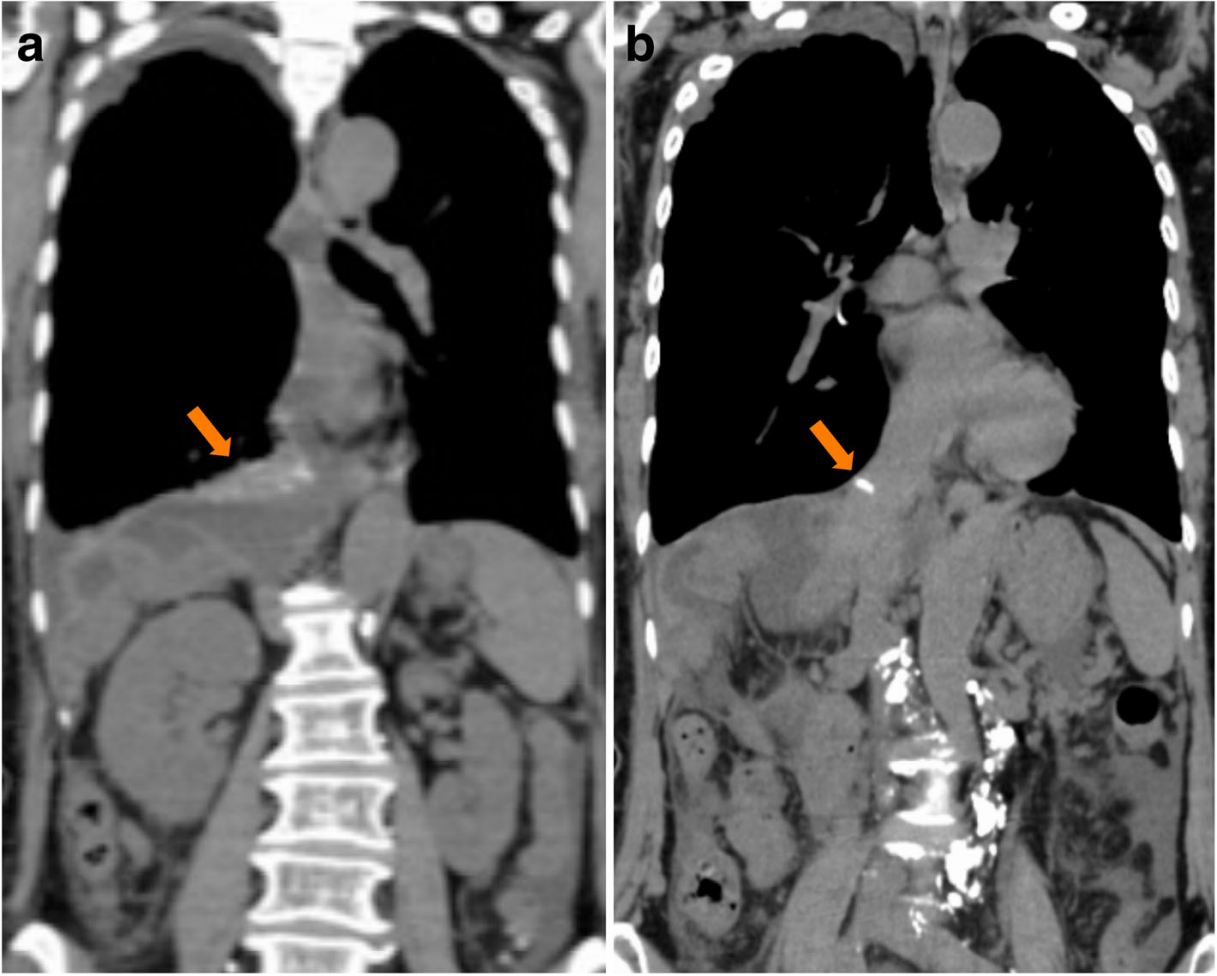
**a** Post-procedure computed tomography revealing extravasation of Lipiodol (ethiodized oil) adjacent to the right mediodorsal pleural space on the diaphragm, but the location of the leak was not identified. The arrow pointing to the extravasation of Lipiodol (ethiodized oil). **b** Computed tomography revealing accumulation of Lipiodol (ethiodized oil) near the staple line of the stump of the right hepatic vein. The arrow pointing to the staple line of the stump of the right hepatic vein

**Fig. 6:**
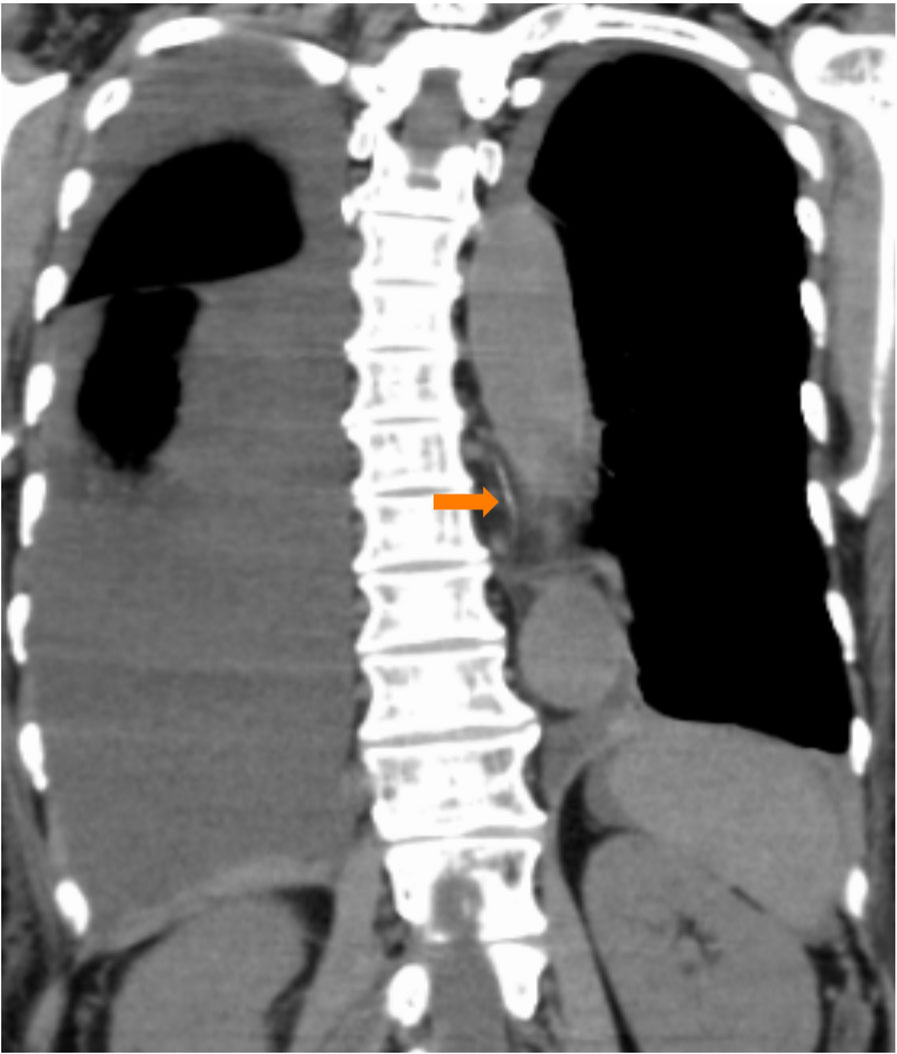
Post-procedure computed tomography after lymphangiography with Lipiodol (ethiodized oil) showing neither obstruction nor dilation of the thoracic duct. The arrow pointing to the thoracic duct

Given that conservative management was ineffective in definitively treating the chyle leak, we performed lymphangiography through the inguinal lymph nodes with Lipiodol (ethiodized oil) on POD 62 to identify the location of the chyle leak and to develop a therapeutic strategy. Lymphangiography and post-procedure CT revealed that there was extravasation of the Lipiodol (ethiodized oil) near the right mediodorsal pleural space along the diaphragm, with an accumulation of Lipiodol (ethiodized oil) located near the staple line of the stump of the right hepatic vein; the exact location of the leak was not identified (Fig. 5). CT also showed that there was neither obstruction nor dilation of the thoracic duct (Fig. 6). An abdominal source of the chyle leak was not demonstrated. A chest tube was placed after the lymphangiography. The tube drained less than 500 mL/day for a week, and the pleural effusion resolved 10 days after the lymphangiography. She was discharged on POD 72. Two and a half years after the original surgery, she is doing well without evidence of recurrence of either chylothorax or cancer.

## Discussion

Here we elucidate two important clinical issues: the possibility of chylothorax after hepatectomy, and the fact that lymphangiography may be effective for both the diagnosis and treatment of this clinical problem.

Many cases of chylothorax occur after surgery. Although postoperative chylothorax usually occurs after thoracic surgery or retroperitoneal surgery, chylothorax after abdominal surgery is extremely rare, and there have been few reports in the literature (Table 1) [2, 13–15]. One report in the literature described a case of chylothorax after donor right hepatectomy for liver transplantation. Right pleural effusion occurred on POD 6 and chylothorax was treated conservatively, without ever identifying the source of the leak. The author discussed that the thoracic duct may have been injured due to its proximity to the surgical area. They also discussed the fact that perioperatively the patient was fasting, which resulted in serous-appearing chyle, making recognition of an intraoperative injury more difficult. However, we did not suspect a thoracic duct injury in their case, because right hepatectomy without lymph node dissection usually does not involve surgery around the thoracic duct. Right hepatectomy does require dissection around the vena cava foramen to divide the right hepatic vein, and we presumed that this was potentially the cause of the chylothorax after hepatectomy. Three other cases in the literature include a total gastrectomy for gastric cancer, a case of upper partial gastrectomy for gastric cancer, and a Nissen fundoplication for a hiatal hernia. These all included dissection around the esophageal hiatus, an area at risk for lymphatic injury, particularly when the dissection extends into the mediastinum adjacent to the thoracic duct. The onset of chylothorax in these cases was from POD 2 to 10. Three cases described a right-sided pleural effusion, and two cases described a left-sided effusion. Thoracoscopy was performed in three cases. However, no cases identified the location of the chyle leak, and all cases improved with drainage and pleurodesis. Two of the cases received TPN. The results of these reports are summarized in Table 1.

**Table 1:** Chylothorax after abdominal digestive surgery

Author	Age	Sex	Primary disease	Operation	POD	Pleural side	Detailed examination	Location of leakage	Treatment
Griffo *et al*., 2010 [15]	59	M	Gastric cancer	Total gastrectomy	2	Right	Thoracoscopy	Not identified	Pleurodesis
Griffo *et al*., 2010 [15]	62	F	Hiatal hernia	Nissen fundoplication	7	Right	Thoracoscopy	Not identified	Pleurodesis
Griffo *et al*., 2010 [15]	65	F	Gastric cancer	Upper partial gastrectomy	5	Right	Thoracoscopy	Not identified	Pleurodesis
van der Vliet *et al*., 1985 [13]	78	F	Gastric cancer	Total gastrectomy	10	Left	No	Not identified	TPN
van der Vliet *et al*., 1985 [13]	66	M	Gastric cancer	Total gastrectomy	4	Left	No	Not identified	TPN
Doerr *et al*., 2005 [2]	–	–	–	Partial gastrectomy	–	–	No	–	–
Li *et al*., 2007 [14]	39	M	Liver donor	Right hepatectomy	6	Right	No	Not identified	Pleural drainage
Our case	74	F	Cholangiocarcinoma	Right hepatectomy	42	Right	Lymph- angiography	Not identified	Lymph- angiography

The case of Doerr *et al*. [2] was not described in detail

*F* female, *M* male, *POD* postoperative day, *TPN* total parenteral nutrition

Initially in our case, chylous ascites occurred in combination with a small right pleural effusion on POD 5 after right hepatectomy. Chylothorax was diagnosed when our patient experienced dyspnea on POD 42, at which point the ascites itself was minimal. Lymphangiography revealed that there was a leak into the pleural space, with no evidence of leak into her abdominal cavity. There was neither obstruction nor dilation of the thoracic duct, and the exact location of the leak in her chest was not identified. We conjectured that the chylothorax could be caused by transdiaphragmatic passage of chylous ascites, in addition to the lymph ducts located in the diaphragm or the sternocostal triangle foramen [16, 17]. However, in our case, the chylothorax was ultimately not due to transdiaphragmatic passage of chylous ascites, because there was no chyle leaking into her abdomen as shown on lymphangiography. Moreover, we did not injure her diaphragm during the mobilization of her liver, and her portal vein was not accessed through her chest and diaphragm for preoperative portal embolization. We inferred that the dissection around the vena cava foramen disrupted fine lymph ducts, resulting in chylous ascites. Eventually, the lymphatic fluid was drawn into the right pleural space by negative intrathoracic pressure, and postoperative intra-abdominal adhesions sealed the vena cava foramen, after which the fluid could not drain into her abdomen but only into the right pleural space. Extravasation of Lipiodol (ethiodized oil) was found adjacent to the right mediodorsal pleural space on the diaphragm, and the accumulation of Lipiodol (ethiodized oil) near the staple line of the stump of the right hepatic vein supported the aforementioned hypothesis. In a review of the literature and the present case, a common feature of chylothorax after abdominal surgery is that the operation involves dissection around the diaphragmatic hiatus.

We also found lymphangiography to be effective for both diagnosis and treatment of chylothorax after hepatectomy. Chest tube drainage and dietary modifications (nil by mouth/TPN) are the standard initial treatments for postoperative chylothorax, and a somatostatin analog may reduce the amount of the thoracic duct lymph flow, leading to earlier resolution of the leak [18, 19]. In our case, these conservative treatments were ineffective. Although the chylous effusion diminished while our patient was on TPN, resumption of an oral diet led to a recurrence of the chylothorax. Thus, we performed lymphangiography through the inguinal lymph nodes using Lipiodol (ethiodized oil) to identify the location of the leak and to potentially develop a therapeutic strategy. Lymphangiography revealed that there was extravasation of Lipiodol (ethiodized oil) into the pleural space without extravasation in the intra-abdominal space; the exact anatomic location of the leak, however, was not identified. There was no evidence of thoracic duct injury. Fortunately, the chylous effusion diminished soon after the lymphangiography, perhaps suggesting that lymphangiography had a therapeutic effect in our case. We presumed that the minor tributaries of the thoracic duct were occluded by the Lipiodol (ethiodized oil), and that subsequent sclerosis of these small lymphatic vessels occurred and the leak stopped. There are some previous case series describing a therapeutic value with lymphangiography alone. One series report described that postoperative chylothorax and chyloperitoneum resolves 64% of the time after this test [20–23]. Chylothorax after thoracic surgery often occurs secondary to injury of the thoracic duct, so the location of the chyle leak could be identified in many cases. However, in contrast to thoracic cases, it is very difficult to identify the location of the leak after abdominal surgery, possibly indicating that a chyle leak after abdominal surgery is due to disruption of small lymphatic channels. In these cases, the Lipiodol (ethiodized oil) that is administered could potentially occlude the small lymphatic ducts and diminish a pleural chylous effusion or chyloperitoneum.

Postoperative pleural effusion is a common complication after hepatectomy, particularly after right-sided resections [24–26]. Most cases are serous effusions but some cases lead to empyema. Pleural drainage is typically performed in cases of dyspnea, fever, or presumed infection. However, chylothorax should be considered when a prolonged pleural effusion develops after hepatectomy. A long period of continuous drainage in the setting of chylothorax is associated with immunodeficiency and malnutrition. Postoperatively, patients who drain more than 1 L/day for 5 to 7 days typically require a surgical intervention, but these observations are based on reports after thoracic surgery [12, 27]. In our case, we continued conservative therapy for 61 days as the general condition of our patient was good. However, she had a delayed recurrence of the chylothorax.

## Conclusions

In conclusion, we present a case of chylothorax after hepatectomy for hilar cholangiocarcinoma. Chylothorax can occur after hepatectomy, and should be considered when a pleural effusion occurs after hepatectomy. Our case suggests that lymphangiography may be effective in the diagnosis and treatment of chylothorax after hepatectomy.

## Abbreviations

CT: Computed tomography
POD: Postoperative day
PTPE: Percutaneous transhepatic portal vein embolization
TPN: Total parenteral nutrition
